# The Galaxy platform for accessible, reproducible and collaborative biomedical analyses: 2018 update

**DOI:** 10.1093/nar/gky379

**Published:** 2018-05-22

**Authors:** Enis Afgan, Dannon Baker, Bérénice Batut, Marius van den Beek, Dave Bouvier, Martin Čech, John Chilton, Dave Clements, Nate Coraor, Björn A Grüning, Aysam Guerler, Jennifer Hillman-Jackson, Saskia Hiltemann, Vahid Jalili, Helena Rasche, Nicola Soranzo, Jeremy Goecks, James Taylor, Anton Nekrutenko, Daniel Blankenberg

**Affiliations:** 1 Department of Biology, Johns Hopkins University, Baltimore, MD, USA; 2 Department of Computer Science, Albert-Ludwigs-University, Freiburg, Freiburg, Germany; 3 Institut Curie, PSL Research University, Paris, France; 4 Department of Biochemistry and Molecular Biology, Penn State University, University Park, PA, USA; 5 Center for Biological Systems Analysis (ZBSA), University of Freiburg, Freiburg, Germany; 6 Department of Pathology, Erasmus Medical Center, Rotterdam, The Netherlands; 7 Department of Biomedical Engineering, Oregon Health and Science University, OR, USA; 8 Earlham Institute, Norwich Research Park, Norwich, UK; 9 Genomic Medicine Institute, Lerner Research Institute, Cleveland Clinic, Cleveland, OH, USA

## Abstract

Galaxy (homepage: https://galaxyproject.org, main public server: https://usegalaxy.org) is a web-based scientific analysis platform used by tens of thousands of scientists across the world to analyze large biomedical datasets such as those found in genomics, proteomics, metabolomics and imaging. Started in 2005, Galaxy continues to focus on three key challenges of data-driven biomedical science: making analyses *accessible* to all researchers, ensuring analyses are completely *reproducible*, and making it simple to *communicate* analyses so that they can be reused and extended. During the last two years, the Galaxy team and the open-source community around Galaxy have made substantial improvements to Galaxy's core framework, user interface, tools, and training materials. Framework and user interface improvements now enable Galaxy to be used for analyzing tens of thousands of datasets, and >5500 tools are now available from the Galaxy ToolShed. The Galaxy community has led an effort to create numerous high-quality tutorials focused on common types of genomic analyses. The Galaxy developer and user communities continue to grow and be integral to Galaxy's development. The number of Galaxy public servers, developers contributing to the Galaxy framework and its tools, and users of the main Galaxy server have all increased substantially.

## INTRODUCTION

Advances in biomedicine and biology increasingly rely on analysis of large datasets. Started in 2005, the Galaxy Project (https://galaxyproject.org) (1–3) maintains a focus on enabling data-driven biomedical science by pursuing three goals: (a) *accessible* data analysis serving all scientists regardless of their informatics expertise and tool developers seeking a wider audience and broad integration of their tools; (b) *reproducible* analyses regardless of the particular platform and (c) *transparent communication* of analyses, which in turn enables reuse and extension of analyses across communities of practice. The Galaxy Project consists of four complementary components:

1. The main public Galaxy server (https://usegalaxy.org)—this server is the subject of this article and has been online since 2007. It features a rich toolset for large-scale genomics analyses, terabytes of public data for use, and hundreds of shared analysis histories, workflows, and interactive publication supplements. This server has more than 124,000 registered users whom run ∼245,000 analysis jobs each month.
2. The Galaxy framework and software ecosystem (https://github.com/galaxyproject)—an open-source software package that anyone can use to run a Galaxy server on any Unix-based operating system. The Galaxy ecosystem includes a software development kit (SDK) for Galaxy tool development, API language bindings for multiple programming languages, software for scripting Galaxy interactions, and tools for automating setup and deployment of Galaxy and its plugins such as tools and visualizations.
3. The Galaxy ToolShed (https://toolshed.g2.bx.psu.edu/)—a community-driven resource for the dissemination of Galaxy tools, workflows, and visualizations. This server functions as an ‘AppStore’ for Galaxy servers where developers and Galaxy admins can host, share, and install Galaxy tools, workflows and visualizations.
4. The Galaxy Community (https://galaxyproject.org/community/)—distinct and complementary subcommunities make key contributions to all aspects of the Project. These subcommunities address the needs and desires of every category of stakeholder including users, administrators, developers, resource providers and educators.

**Figure 1.**
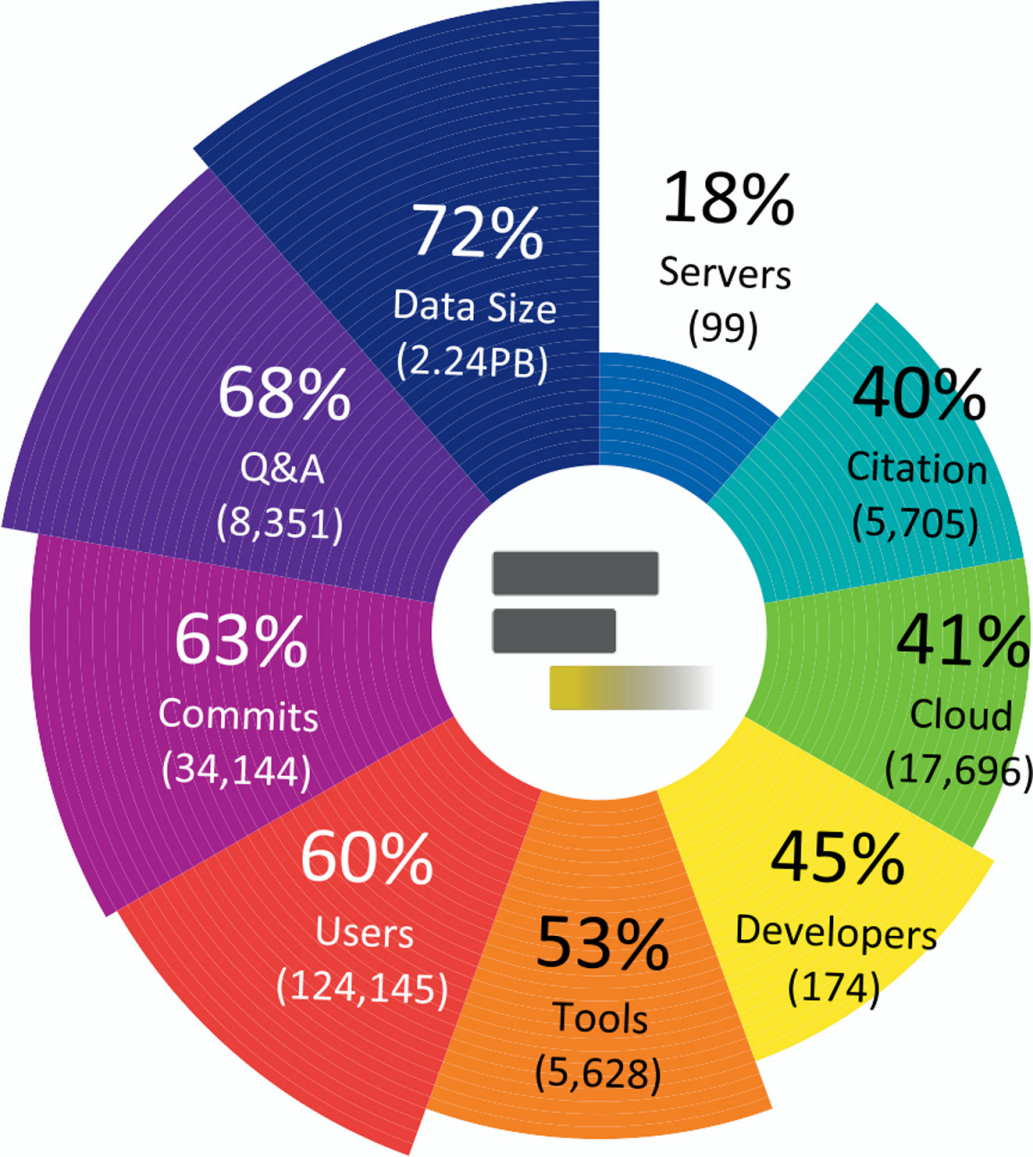
Circular barplot illustrating recent growth of the Galaxy Project across several independent facets. In the past two years, usage of the main public Galaxy server has increased 60%, the number of tools and supported versions has increased 53%, and the amount of data analyzed on the main server has increased 72%. A growing number of public instances (18% increase) and cloud-based Galaxy instances (38% increase) provide researchers with a wider range of options for scalability and application domains. Additionally, more developers (45% increase with 63% more commits to the codebase) contributed to the Galaxy framework and software ecosystem. Question and answer activity on the Galaxy Biostars forum increased 68%.

Galaxy has served hundreds of thousands of users, been used in >5700 scientific publications, and provided 500+ developers with a framework provisioning accessible, transparent, and reproducible data analysis (https://galaxyproject.org/galaxy-project/statistics/). Many instances of the framework have been installed, including Galaxy *Main* (https://usegalaxy.org) and over 99 publicly accessible servers (https://galaxyproject.org/public-galaxy-servers/), serving biomedical and other domain-specific research. Significant growth has occurred across all sectors of the Galaxy Project within the past two years (Figure 1).

## NEW FEATURES

### Scalability

Scalability is amongst the most significant challenges that Galaxy faces as the size and number of biomedical and especially genomics datasets continues to grow. For instance, single-cell RNA-seq experiments routinely generate hundreds or thousands of primary datasets. As a web-based application, Galaxy must scale both in its web-based interface and on its backend server and do so in a multiuser environment.

*User interface scalability* enables scientists to use the Galaxy web interface to analyze *many* datasets, apply (collective) operations on them, and design pipelines to analyze them. Galaxy implements a variety of features to facilitate analyzing large numbers of datasets, including *workflows* and *collections*. Our recent optimizations of the user interface (UI) yielded a significant improvement to frontend scalability. We benchmarked the optimizations by replicating an experiment conducted on single Hematopoietic stem cells and multipotent progenitors (4) to quantify the expression of 64 000 transcripts, which generates 11 872 history items. Galaxy ran this proof of concept experiment seamlessly using existing standard tools, whereas earlier versions of Galaxy would not have been able to support this analysis.

**Figure 2.**
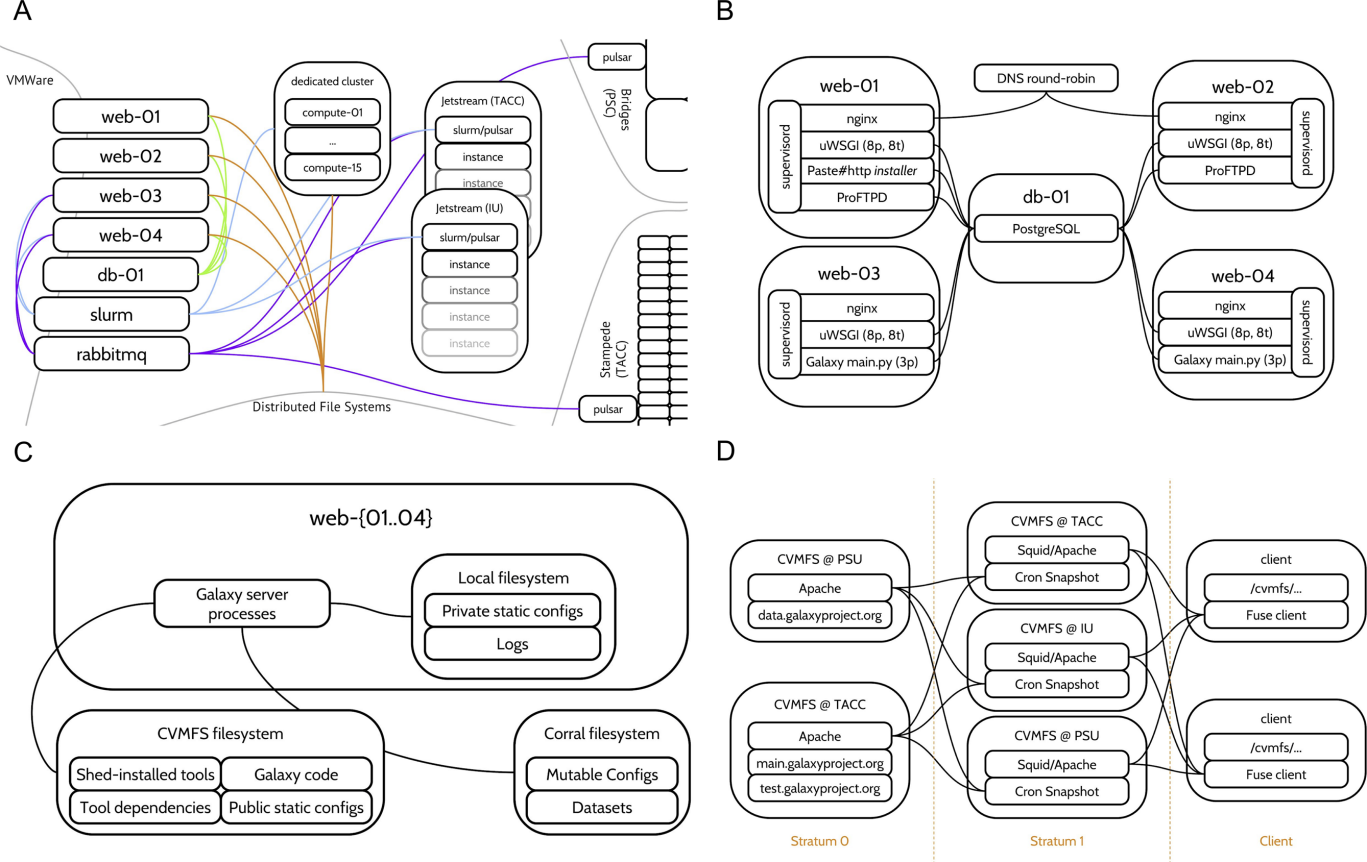
Schematic of servers and services in use at Galaxy Main. (**A**) A global overview of Galaxy Main resources. When users interact with usegalaxy.org, their browser connects to one of two frontends (shown as web-01/02) with file uploads being handled by web-03/04; each of these web servers connects to a database server and mounts a set of shared distributed file systems. Web-03/04 also prepares and schedules jobs using Slurm directly to manage compute tasks on fifteen dedicated compute nodes, which also directly mount the shared distributed file systems. A combination of Slurm and Pulsar (https://github.com/galaxyproject/pulsar) are used to manage tasks and for dataset file staging, respectively, on the Jetstream cloud at Indiana University (IU) and the Texas Advanced Computing Center (TACC). Communication between Galaxy and Pulsar is handled using the RabbitMQ (https://www.rabbitmq.com/) message broker. Additional jobs are sent to the supercomputer systems Bridges at Pittsburgh Supercomputing Center (PSC) and Stampede at TACC using Pulsar. These various compute resources are chosen based upon tool and job characteristics. See, e.g. https://github.com/galaxyproject/usegalaxy-playbook/wiki/Infrastructure for specific and up-to-date information. (**B**) Multiple frontend servers provide Galaxy content to users by utilizing round-robin load balancing. Nginx (https://nginx.org/) is used to serve HTTP content from the Galaxy uWSGI web application. Individual software processes are monitored and controlled using Supervisor (http://supervisord.org/). Each of these frontend servers connects to a PostgreSQL (https://www.postgresql.org/) database server. (**C**) Layout of data schemes used by Galaxy Main is optimized for application speed, concurrent access, and versioned content. Each Galaxy frontend server utilizes a combination of shared distributed file systems, CVMFS for versioned semi-static content and TACC’s Corral filesystem via NFS for mutable content, along with server-specific local file systems. (D) CernVM File System (CVMFS) infrastructure hosted by the Galaxy Project that is used at Main and available for access to any other Galaxy instance. Stratum 0 contains the single-source modifiable data repositories. File content is served using the Apache HTTP server (https://httpd.apache.org/). To enable redundancy and scaling to a large number of clients, Stratum 1 replica servers are hosted at multiple locations and utilize Squid (http://www.squid-cache.org/) for data caching. Additional replica servers can also be hosted by community members. Individual clients (Galaxy instances and compute nodes) access data content from Stratum 1 servers using a Filesystem in Userspace (FUSE) mount.

**Figure 3.**
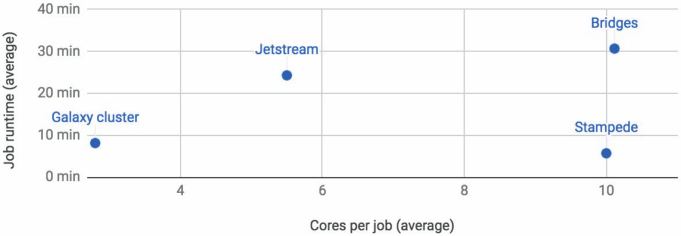
Enabling automated selection and use of specialized national cyberinfrastructure compute resources from Galaxy Main enhances user-experience. It is now possible to run jobs that are up to an order of magnitude larger than before by using Bridges and Stampede. New types of jobs, such as interactive environments (see *Advances in tools* section), that require execution isolation due to security concerns are enabled by utilizing virtualization facilitated by the Jetstream cloud. Consequently, it is possible to concurrently run more jobs due to the increase in processing capacity.

*Server scalability* refers to the Galaxy's ability to execute *many* data analysis/manipulation tasks for *many* users. This is achieved by advantageously utilizing a range of available computing resources. The Galaxy framework runs on various platforms, from a standard laptop to institutional clusters and cloud-based platforms. Galaxy is highly versatile in its ability to deploy jobs (atomic units of work), as it can leverage a multitude of workload managers including Slurm (5), HTCondor (6), Apache Mesos (7) and Kubernetes (https://kubernetes.io), among others, in addition to a built-in lightweight job running system. Recent enhancements to Galaxy's job management include dynamic job destination assignment (which facilitate automatic job parameter-specific resource selection), delay in job queuing (e.g. for workflows), automatic job re-submission (e.g., on job failure due to a temporary cluster error), and means of implementing fair-share prioritization schemes. These features are being used on Galaxy Main (Figure 2) to leverage cloud computing resources for better job throughput. Specifically, Galaxy Main is now configured to take advantage of the XSEDE infrastructure (8) that includes Bridges and Stampede resources as well as the Jetstream cloud (9). The benefits of using these resources include the ability to run larger jobs, as shown in Figure 3. Additionally, use of these resources has enabled new types of analysis to be enabled on Main. Notably, this includes Galaxy Interactive Environments through the ability to use containerization technologies and provide sufficient isolation of individual jobs from other processes running on the same underlying compute infrastructure.

A complete Galaxy server with a full repertoire of tools and reference data can be run on major cloud platforms. These servers are launched independently by users, and come pre-configured with hundreds of tools and reasonable default settings typical of a production server. Notably, launched instances do not have usage quotas and can be customized to install any desired tool. We have designed a cloud-agnostic approach for leveraging these resources by developing the abstraction library CloudBridge (10) and a new CloudLaunch application. These two solutions make it possible to launch Galaxy instances across a variety of cloud providers while reducing the requirement to build and maintain cloud-specific resources (e.g. machine images, file systems). There are now 10 different flavors of Galaxy available for launching on major clouds including Amazon Web Services, Jetstream and Microsoft Azure (https://launch.usegalaxy.org).

### Advances in tools

The Galaxy ToolShed (11) assumes the role of an AppStore for Galaxy instances by hosting thousands of tools. The ToolShed improves tool availability, deployment, and portability across Galaxy servers and computing environments.

#### Updated tool suite

Over the last two years, we have expanded both the quantity and quality of the tools available on the Galaxy ToolShed. As of April 2018, the ToolShed hosts 5628 tools, which shows 53% growth since 2016, and ∼2000 repositories had at least one new update. Examples of new tools include: GEMINI for exploring genetic variation (12); mothur for analyzing rRNA gene sequences (13); QIIME for quantitative microbiome analysis from raw DNA sequencing data (14); deepTools for explorative analysis of deeply sequence data (15,16); HiCexplorer (17) for analysis and visualization of Hi-C data; ChemicalToolBox for comprehensive access to cheminformatics libraries and drug discovery tools (18); minimap2 (https://arxiv.org/abs/1708.01492) and poretools for long read sequencing analysis (19); MultiQC (20) to aggregate multiple results into a single report; a new RNA-seq analysis tool suite with modern analysis tools such as Kallisto (21), Salmon (22), Deseq2 (23) and STAR-Fusion (24), and GenomeSpace (25), a cloud-based interoperability tool.

#### Tool environment and interface

The portability and backward-compatibility of the Galaxy tools environment is improved significantly. Accordingly, a tool configuration now includes a *tool profile version*, which is used to ensure compatibility between a version of a tool and its targeted Galaxy version. In addition, tool profile versions allow for the evolution of new and better tool defaults and behaviors while maintaining backwards compatibility. We also improved the ToolShed API and its interface to facilitate installing tools missing from an imported workflow. We improved the installation process so that restarting Galaxy is not required to use a newly installed tool.

### Interactive analysis and visualization

Galaxy's UI makes it possible for anyone to run complex analyses. However, a complete analysis of genomic data often requires custom scripts or visualizations, especially at the beginning (data preparation) or end (data summarization) of analyses. To meet these customized needs, we recently introduced Galaxy Interactive Environments (26), an integration of Galaxy with Jupyter (RStudio is in development)—a commonly used interactive scripting platform. With Interactive Environments, Galaxy users benefit from existing computational infrastructure via both graphical UI and ad hoc scripting, or any combination of these.

Galaxy's visualization framework (27) makes it possible to integrate a wide variety of Web-based and server-side visualizations. Through this framework, many new visualizations have been added to Galaxy, including Cytoscape (28), and the WebGL enabled 3D Protein viewer NGL (29), molecular interaction networks and macromolecular structures visualizations, and the 100+ visualizations available through BioJS (30), a rich set of community-driven JavaScript components for agile and interactive visualization of biological data.

### User interface and experience enhancements

There are two common modes of data analysis: exploratory and pipeline execution. Galaxy enables simultaneous access to both of these. Users are able to interactively analyze their data by making use of individual tools in a trial-and-error manner. They are then able to automatically generate reusable and generalizable workflows from an ad hoc analysis. An interactive workflow editor is also available to modify or generate workflows from scratch. At any point in time, a user can seamlessly switch modes between interactively analyzing datasets and executing a workflow on these datasets. There is no analysis lock-in, and users can exercise full control, or make use of pre-existing pipelines. Importantly, these analysis artefacts, such as datasets, analysis histories, workflows, and visualizations can all be shared and copied by collaborators at the discretion of the analyst.

#### Client-side infrastructure

The client-side of Galaxy, which is the user-interface most people associate with Galaxy, has seen significant changes under the hood. The usage of server-side mako templates, for example to create forms, has been further reduced and replaced by client-side only code that communicates via the RESTful Galaxy API with the backend. This minimizes the number of full-page refreshes and improves response time by enabling partial page updates. The interface has been further enhanced to allow for drag-and-drop of files and datasets, presents a fuzzy search on dataset and tool metadata, and implements a modal scratchbook for visualizations and comparison of multiple datasets.

Furthermore, the community has selected the Vue.js framework (https://vuejs.org/) as the base for future improvements allowing all UI elements to converge into a more reactive and future-proof interface. With the integration of Vue.js, the entire client-side build system was updated to utilize the latest web-technologies, to make routing and loading times faster, and to encourage rapid future interface improvements. While mostly transparent to users, these changes are the fundamental groundwork of a much more flexible UI framework that will enable visual enhancements and an improved user experience for years to come.

#### Tags

Although tags have been supported in Galaxy for several years, they have only recently become advantageous for large many-sample analyses. We have enhanced tags to allow propagation through dataset analysis steps. This facilitates tracking individual datasets through the entire analysis life-cycle and becomes part of the provenance system and ease-of-use of Galaxy. To enable automatic tag propagation, a hash-sign (#) is placed at the beginning of the tag, which is colloquially referred to as a named-tag. While standard Galaxy output dataset naming is suitable for many interactive analyses, the connection between inputs and outputs through large workflows becomes increasingly less obvious; by utilizing named-tags, users can label datasets with an identifier that is maintained throughout the analysis.

#### Webhooks

Inspired by user feedback and the need to quickly modify and adapt Galaxy's interface, we integrated a pluggable system to extend Galaxy's frontend. Webhooks provide an entry-point into the Galaxy UI, in which it is possible to add buttons, menu entries, or entire iframes. At these entry-points a developer can dynamically add client-side code (JavaScript, HTML, CSS) and interact with the rest of the Galaxy user-interface. By integrating Webhooks with the Galaxy API, it is also possible to trigger server-side functions from within a Webhook. Webhooks can be thoroughly customized and are enabled at the discretion of the Galaxy administrator.

#### Interactive tours

We have developed self-paced, interactive tours that users can step through to learn about Galaxy. These tours guide users step by step through using the interface including tools, workflows, and other features available in Galaxy. To simplify tour creation, a Tour Builder (https://github.com/TailorDev/galaxy-tourbuilder) has been created for recording, replaying, updating and exporting tours.

#### Improved workflows

Galaxy workflows have been extended in several ways. Switching between tool versions and upgrading workflows with new tool versions is now supported. A workflow can now be embedded in another, making it easier to create and edit workflows that have many common steps repeated. Many of these features have existed in in standalone workflow systems, such as Taverna (31), for sometime, but have been widely requested by Galaxy users. Workflows are now scheduled by a Galaxy server more efficiently and in the background, making it possible to execute larger workflows, generating tens of thousands of jobs, while providing instant feedback and a snappier user-experience. We have also enhanced Galaxy with initial support for running workflows defined in the Common Workflow Language (32) format.

#### Dataset collections

Galaxy Dataset Collections combine datasets to enable simultaneous analysis. They organize sets of datasets as potentially nested lists of objects allowing easier data handling and batch execution of tools. In addition to the related frontend improvements, and support of nesting collections together, we recently introduced specialized tools to be executed on collections (e.g. *Collapse*, which combines a list of datasets into a single dataset, *Flatten* which takes nested collections and produces a flat list of datasets, and *Merge* which takes two lists and creates a single unified list), and enabled uploading and downloading dataset collections to and from both user's local disk and Galaxy data libraries.

### Infrastructure enhancements

In order to make Galaxy more robust in a production environment, we adopted technologies to enhance Galaxy's portability, security, reliability, and scalability. Galaxy now utilizes uWSGI (http://projects.unbit.it/uwsgi) as its default web application server. This adoption has several advantages, namely the ability to negate Python's limitations regarding concurrent tasks execution, built-in load balancing, scalability, improved fault tolerance and the possibility of restarting Galaxy uninterruptedly.

Many tools available via Galaxy rely on the availability of reference and index data. To promote ease of use and efficient storage and compute resources, Galaxy is able to share a precomputed set of local reference data for tools to use. Previously, making this data available to the tools was a time intensive process where a Galaxy administrator had to install and properly configure the server, either manually or by using Data Managers (33). However, this resulted in much redundant effort required for each Galaxy server being configured. To streamline this process, we have made all the reference data we prepared for Galaxy Main available via a CernVM File System (CVMFS; (34)), a scalable and content-addressable file system. This repository currently hosts 5TB of pre-build reference data, which are versioned and shared publicly with read-only access. With minimal configuration, any instance of Galaxy, including Galaxy-Docker images, can attach to this file system and gain access to the same reference data available on Galaxy Main. To improve accessibility and fault-tolerance, this data source is replicated on servers located in Europe and Australia.

Galaxy is powered by various open-source projects which are installed automatically, and used when needed. Galaxy is using the Conda package manager (https://conda.io) as its default tool dependency resolver, and offers support for virtualization and containerization technologies (e.g. Docker (https://www.docker.com) and Singularity (35)) to ensure a higher level of portability, if needed. By leveraging the Bioconda (https://doi.org/10.1101/207092) and the BioContainer (36) projects, Galaxy is able to provision and use reproducible tool execution environments ((37); https://doi.org/10.1101/200683).

Galaxy is a generic data analysis framework, which can be configured for various application scenarios using a wide range of configuration parameters. To facilitate configuring these parameters with optimal values for a number of predefined application scenarios, the Galaxy project leverages Ansible (https://www.ansible.com), software for automated configuration and management of other software packages. We have developed and shared Ansible configurations for Galaxy *Main*, the main public Galaxy server, (https://github.com/galaxyproject/usegalaxy-playbook) and also a configurable generic playbook for setting up production instances on cloud resources, virtual machines, and bare metal (https://github.com/ARTbio/GalaxyKickStart). This playbook can be used as a reference for configuring a Galaxy instance for a production environment.

The Galaxy-Docker project (https://github.com/bgruening/docker-galaxy-stable), delivers a production ready Galaxy instance in minutes and can be used as the basis for personalized, self-contained, portable instances of Galaxy, known as Galaxy flavors. Preconfigured by the Galaxy community, a plenitude of flavors already exist covering application scenarios, from BLAST+ (38,39), metagenomics (https://doi.org/10.1101/183970), ChIP-exo analysis, or RNA research (40). In addition to the facilitated and out-of-box functionality, these images provision isolated environments well-suited for experimenting with tools and Galaxy configurations, and are ideal for training courses, as demonstrated by the Galaxy Training Network.

Server monitoring and issue management is crucial in production Galaxy instances. Galaxy has integrated a plugin module to submit user bug-reports to configurable endpoints such as mailing lists or GitHub issues. With this, Galaxy can be configured to send error reports to a local ticket system. The recent integration of Sentry (https://sentry.io/) for automated error tracking and reporting makes it easier for administrators to track both client- and server-side errors without requiring manual user bug reports.

## COMMUNITY

Galaxy serves several distinct communities: researchers, tool developers, resource providers, trainers, and trainees. To centralize resources for all communities, we have developed the Galaxy Community Hub (https://galaxyproject.org) for all things Galaxy. The Hub uses a modified wiki approach, with content written in Markdown, a simple formatting language, and then built into a static website. Anyone can update the Markdown documents using GitHub pull requests, a standard approach for collaborating on code and documentation on GitHub projects. Submitted pull requests are reviewed and merged, and the Hub site is automatically regenerated and updated, resulting in high-quality reviewed content that can be updated by any member of the Galaxy community. The Hub includes a full list of public Galaxy servers (https://galaxyproject.org/public-galaxy-servers), a large set of tutorials for learning to use Galaxy and perform genomic analyses, extensive documentation on deploying and administering a Galaxy server in the Cloud or on local hardware, and upcoming events. We also maintain an annotated listing of the >5000 publications referencing Galaxy via the free and open-source Zotero service (https://www.zotero.org/groups/1732893/galaxy).

The Main Galaxy server has over 124 000 registered users and ∼2000 new users register each month. On average, 20 000 unique users execute over 245 000 analysis jobs by accessing 750 different tools every month. With such an active user-base, questions on platform and tool usage, as well as general research questions (41), are common. To efficiently assist users in performing research, we provide a Biostars (42) Question and Answers forum (https://biostar.usegalaxy.org/) that leverages the knowledge and strength of community members to provide support. This forum is monitored and moderated by core team members, but the Galaxy user community provides many answers. Help is also available through live chat with the team and community members via Gitter and IRC chat services, which are used most often by developers and administrators. In addition to the online help and documentation, the Galaxy Training Network has developed comprehensive tutorials and workflows for performing common data analysis tasks, providing topic-specific introduction slides, hands-on material, sample data, and even playable Galaxy tours (https://doi.org/10.1101/225680).

Many in-person events that highlight and build the Galaxy community occur each year (https://galaxyproject.org/events/). These include free or low-cost hands-on workshops and training sessions that have been hosted by the community on six continents. The Galaxy Community Conference (GCC) is an annual conference that was first held in 2010. GCC alternates between Europe and the United States, includes two full days of training, two days of coding and data analysis hackathons, and two days of oral and poster presentations. Galaxy conferences have had over two hundred attendees each year since 2012, and over eleven hundred different researchers have attended since 2010. Our 2018 conference will be hosted jointly with the Bioinformatics Open Source Conference (BOSC) in an effort to promote and centralize discussion of open-source software for bioinformatics.

Another core area of community focus is tool development and availability. The Intergalactic Utilities Commission (IUC; https://galaxyproject.org/iuc/) is a community-based organization that defines best-practices for tool development that help ensure the availability of high-quality tools in the ToolShed. It is a self-organizing and self-regulating group that has grown by six new members in the last two years and is primarily composed of individuals outside of the core Galaxy development team. The IUC is only one of many tool contributors, with the ToolShed allowing any member of the community to share tools that they have added to Galaxy. To assist community members with tool development and distribution, a command-line tool named Planemo (https://github.com/galaxyproject/planemo) has been developed. Planemo provides functionality for verifying best-practice adherence, testing, installation and uploading of tools to the ToolShed.

Community contributions have helped the Galaxy framework and its tool suite to grow considerably. One hundred and seventy-four developers, who have collectively produced 13 135 commits within just the past two years (63% increase since January 2016), have improved Galaxy's scalability, functionality, and usability. The project utilizes the Travis and Jenkins continuous integration (CI) services to automatically execute comprehensive test suites on each set of proposed code changes. This strategy helps prevent the introduction of bugs to the codebase and improves review time. By harnessing the open-source community and modern software development practices, we are able to release a new stable version of the Galaxy framework every four months. Current future directions include enabling data and compute federation; tighter coupling of Interactive Environments with provenance and reuse; ToolShed installation and development enhancements; continued work on collections, workflows, analysis interfaces and history views; additional training material; improving statistical usage tracking and instrumentation; and much more. For anyone interested in getting involved with Galaxy development, we invite them to read the project's Contributing and Code of Conduct documents, review open issues, and explore the current roadmap, all which are available from the Galaxy GitHub repository (https://github.com/galaxyproject/galaxy/).

## References

[1] Giardine B., Riemer C., Hardison R.C., Burhans R., Elnitski L., Shah P., Zhang Y., Blankenberg D., Albert I., Taylor J. Galaxy: a platform for interactive large-scale genome analysis. Genome Res. 2005; 15:1451–1455. 16169926 10.1101/gr.4086505 PMC1240089

[2] Blankenberg D., Taylor J., Schenck I., He J., Zhang Y., Ghent M., Veeraraghavan N., Albert I., Miller W., Makova K.D. A framework for collaborative analysis of ENCODE data: making large-scale analyses biologist-friendly. Genome Res. 2007; 17:960–964. 17568012 10.1101/gr.5578007 PMC1891355

[3] Afgan E., Baker D., van den Beek M., Blankenberg D., Bouvier D., Čech M., Chilton J., Clements D., Coraor N., Eberhard C. The Galaxy platform for accessible, reproducible and collaborative biomedical analyses: 2016 update. Nucleic Acids Res. 2016; 44:W3–W10. 27137889 10.1093/nar/gkw343 PMC4987906

[4] Yang J., Tanaka Y., Seay M., Li Z., Jin J., Garmire L.X., Zhu X., Taylor A., Li W., Euskirchen G. Single cell transcriptomics reveals unanticipated features of early hematopoietic precursors. Nucleic Acids Res. 2017; 45:1281–1296. 28003475 10.1093/nar/gkw1214 PMC5388401

[5] Yoo A.B., Jette M.A., Grondona M. SLURM: Simple Linux Utility for Resource Management. Job Scheduling Strategies for Parallel Processing, Lecture Notes in Computer Science. 2003; Berlin, Heidelberg Springer 44–60.

[6] Thain D., Tannenbaum T., Livny M. Distributed computing in practice: the Condor experience. Concurr. Comput. 2005; 17:323–356.

[7] Hindman B., Konwinski A., Zaharia M., Ghodsi A., Joseph A.D., Katz R., Shenker S., Stoica I. Mesos: A Platform for Fine-grained Resource Sharing in the Data Center. Proceedings of the 8th USENIX Conference on Networked Systems Design and Implementation. 2011; Berkeley USENIX Association 295–308.NSDI’11.

[8] Towns J., Cockerill T., Dahan M., Foster I., Gaither K., Grimshaw A., Hazlewood V., Lathrop S., Lifka D., Peterson G.D. XSEDE: accelerating scientific discovery. Comput. Sci. Eng. 2014; 16:62–74.

[9] Stewart C.A., Cockerill T.M., Foster I., Hancock D., Merchant N., Skidmore E., Stanzione D., Taylor J., Tuecke S., Turner G. Jetstream: a self-provisioned, scalable science and engineering cloud environment. Proceedings of the 2015 XSEDE Conference: Scientific Advancements Enabled by Enhanced Cyberinfrastructure. 2015; NY ACM 29 XSEDE ’15.

[10] Goonasekera N., Lonie A., Taylor J., Afgan E. CloudBridge: a Simple Cross-Cloud Python Library. Proceedings of the XSEDE16 Conference on Diversity, Big Data, and Science at Scale. 2016; Miami ACM 37. 10.1145/2949550.2949648 PMC8375622 34423340

[11] Blankenberg D., Von Kuster G., Bouvier E., Baker D., Afgan E., Stoler N., Taylor J., Nekrutenko A. Galaxy Team Dissemination of scientific software with Galaxy ToolShed. Genome Biol. 2014; 15:403. 25001293 10.1186/gb4161 PMC4038738

[12] Paila U., Chapman B.A., Kirchner R., Quinlan A.R. GEMINI: integrative exploration of genetic variation and genome annotations. PLoS Comput. Biol. 2013; 9:e1003153. 23874191 10.1371/journal.pcbi.1003153 PMC3715403

[13] Schloss P.D., Westcott S.L., Ryabin T., Hall J.R., Hartmann M., Hollister E.B., Lesniewski R.A., Oakley B.B., Parks D.H., Robinson C.J. Introducing mothur: open-source, platform-independent, community-supported software for describing and comparing microbial communities. Appl. Environ. Microbiol. 2009; 75:7537–7541. 19801464 10.1128/AEM.01541-09 PMC2786419

[14] Caporaso J.G., Kuczynski J., Stombaugh J., Bittinger K., Bushman F.D., Costello E.K., Fierer N., Peña A.G., Goodrich J.K., Gordon J.I. QIIME allows analysis of high-throughput community sequencing data. Nat. Methods. 2010; 7:335–336. 20383131 10.1038/nmeth.f.303 PMC3156573

[15] Ramírez F., Dündar F., Diehl S., Grüning B.A., Manke T. deepTools: a flexible platform for exploring deep-sequencing data. Nucleic Acids Res. 2014; 42:W187–W191. 24799436 10.1093/nar/gku365 PMC4086134

[16] Ramírez F., Ryan D.P., Grüning B., Bhardwaj V., Kilpert F., Richter A.S., Heyne S., Dündar F., Manke T. deepTools2: a next generation web server for deep-sequencing data analysis. Nucleic Acids Res. 2016; 44:W160–W165. 27079975 10.1093/nar/gkw257 PMC4987876

[17] Ramírez F., Bhardwaj V., Arrigoni L., Lam K.C., Grüning B.A., Villaveces J., Habermann B., Akhtar A., Manke T. High-resolution TADs reveal DNA sequences underlying genome organization in flies. Nat. Commun. 2018; 9:189. 29335486 10.1038/s41467-017-02525-w PMC5768762

[18] Lucas X., Grüning B.A., Günther S. ChemicalToolBoX and its application on the study of the drug like and purchasable space. J. Cheminform. 2014; 6:P51.

[19] Loman N.J., Quinlan A.R. Poretools: a toolkit for analyzing nanopore sequence data. Bioinformatics. 2014; 30:3399–3401. 25143291 10.1093/bioinformatics/btu555 PMC4296151

[20] Ewels P., Magnusson M., Lundin S., Käller M. MultiQC: summarize analysis results for multiple tools and samples in a single report. Bioinformatics. 2016; 32:3047–3048. 27312411 10.1093/bioinformatics/btw354 PMC5039924

[21] Bray N.L., Pimentel H., Melsted P., Pachter L. Near-optimal probabilistic RNA-seq quantification. Nat. Biotechnol. 2016; 34:525–527. 27043002 10.1038/nbt.3519

[22] Patro R., Duggal G., Love M.I., Irizarry R.A., Kingsford C. Salmon provides fast and bias-aware quantification of transcript expression. Nat. Methods. 2017; 14:417–419. 28263959 10.1038/nmeth.4197 PMC5600148

[23] Love M.I., Huber W., Anders S. Moderated estimation of fold change and dispersion for RNA-seq data with DESeq2. Genome Biol. 2014; 15:550. 25516281 10.1186/s13059-014-0550-8 PMC4302049

[24] Dobin A., Davis C.A., Schlesinger F., Drenkow J., Zaleski C., Jha S., Batut P., Chaisson M., Gingeras T.R. STAR: ultrafast universal RNA-seq aligner. Bioinformatics. 2013; 29:15–21. 23104886 10.1093/bioinformatics/bts635 PMC3530905

[25] Qu K., Garamszegi S., Wu F., Thorvaldsdottir H., Liefeld T., Ocana M., Borges-Rivera D., Pochet N., Robinson J.T., Demchak B. Integrative genomic analysis by interoperation of bioinformatics tools in GenomeSpace. Nat. Methods. 2016; 13:245–247. 26780094 10.1038/nmeth.3732 PMC4767623

[26] Grüning B.A., Rasche E., Rebolledo-Jaramillo B., Eberhard C., Houwaart T., Chilton J., Coraor N., Backofen R., Taylor J., Nekrutenko A. Jupyter and Galaxy: easing entry barriers into complex data analyses for biomedical researchers. PLoS Comput. Biol. 2017; 13:e1005425. 28542180 10.1371/journal.pcbi.1005425 PMC5444614

[27] Goecks J., Eberhard C., Too T., Nekrutenko A., Taylor J. Galaxy Team Web-based visual analysis for high-throughput genomics. BMC Genomics. 2013; 14:397. 23758618 10.1186/1471-2164-14-397 PMC3691752

[28] Shannon P., Markiel A., Ozier O., Baliga N.S., Wang J.T., Ramage D., Amin N., Schwikowski B., Ideker T. Cytoscape: a software environment for integrated models of biomolecular interaction networks. Genome Res. 2003; 13:2498–2504. 14597658 10.1101/gr.1239303 PMC403769

[29] Rose A.S., Hildebrand P.W. NGL Viewer: a web application for molecular visualization. Nucleic Acids Res. 2015; 43:W576–W579. 25925569 10.1093/nar/gkv402 PMC4489237

[30] Gómez J., García L.J., Salazar G.A., Villaveces J., Gore S., García A., Martín M.J., Launay G., Alcántara R., Del-Toro N. BioJS: an open source JavaScript framework for biological data visualization. Bioinformatics. 2013; 29:1103–1104. 23435069 10.1093/bioinformatics/btt100 PMC3624812

[31] Wolstencroft K., Haines R., Fellows D., Williams A., Withers D., Owen S., Soiland-Reyes S., Dunlop I., Nenadic A., Fisher P. The Taverna workflow suite: designing and executing workflows of Web Services on the desktop, web or in the cloud. Nucleic Acids Res. 2013; 41:W557–W561. 23640334 10.1093/nar/gkt328 PMC3692062

[32] Amstutz P., Crusoe M.R., Tijanić N., Chapman B., Chilton J., Heuer M., Kartashov A., Leehr D., Ménager H., Nedeljkovich M. Common Workflow Language, v1.0. figshare. 2016; https://doi.org/10.6084/m9.figshare.3115156.v2.

[33] Blankenberg D., Johnson J.E., Taylor J., Nekrutenko A. Galaxy Team Wrangling Galaxy's reference data. Bioinformatics. 2014; 30:1917–1919. 24585771 10.1093/bioinformatics/btu119 PMC4071198

[34] Blomer J., Buncic P., Charalampidis I., Harutyunyan A., Larsen and D., Meusel R. Status and future perspectives of CernVM-FS. J. Phys. Conf. Ser. 2012; 396:052013.

[35] Kurtzer G.M., Sochat V., Bauer M.W. Singularity: Scientific containers for mobility of compute. PLoS One. 2017; 12:e0177459. 28494014 10.1371/journal.pone.0177459 PMC5426675

[36] da Veiga Leprevost F., Grüning B.A., Alves Aflitos S., Röst H.L., Uszkoreit J., Barsnes H., Vaudel M., Moreno P., Gatto L., Weber J. BioContainers: an open-source and community-driven framework for software standardization. Bioinformatics. 2017; 33:2580–2582. 28379341 10.1093/bioinformatics/btx192 PMC5870671

[37] Nekrutenko A., Goecks J., Taylor J., Blankenberg D. Galaxy Team Biology needs evolutionary software tools: Let's build them right. Mol. Biol. Evol. 2018; https://doi.org/10.1093/molbev/msy084. 10.1093/molbev/msy084 PMC5967460 29688462

[38] Cock P.J.A., Chilton J.M., Grüning B., Johnson J.E., Soranzo N. NCBI BLAST+ integrated into Galaxy. Gigascience. 2015; 4:39. 26336600 10.1186/s13742-015-0080-7 PMC4557756

[39] Camacho C., Coulouris G., Avagyan V., Ma N., Papadopoulos J., Bealer K., Madden T.L. BLAST+: architecture and applications. BMC Bioinformatics. 2009; 10:421. 20003500 10.1186/1471-2105-10-421 PMC2803857

[40] Grüning B.A., Fallmann J., Yusuf D., Will S., Erxleben A., Eggenhofer F., Houwaart T., Batut B., Videm P., Bagnacani A. The RNA workbench: best practices for RNA and high-throughput sequencing bioinformatics in Galaxy. Nucleic Acids Res. 2017; 45:W560–W566. 28582575 10.1093/nar/gkx409 PMC5570170

[41] Blankenberg D., Taylor J., Nekrutenko A. Online resources for genomic analysis using high-throughput sequencing. Cold Spring Harb. Protoc. 2015; 2015:324–335. 25655493 10.1101/pdb.top083667

[42] Parnell L.D., Lindenbaum P., Shameer K., Dall’Olio G.M., Swan D.C., Jensen L.J., Cockell S.J., Pedersen B.S., Mangan M.E., Miller C.A. BioStar: an online question & answer resource for the bioinformatics community. PLoS Comput. Biol. 2011; 7:e1002216. 22046109 10.1371/journal.pcbi.1002216 PMC3203049

